# A wavelength-convertible quantum memory: Controlled echo

**DOI:** 10.1038/s41598-018-28776-1

**Published:** 2018-07-16

**Authors:** Byoung S. Ham

**Affiliations:** School of Electrical Engineering and Computer Science, Gwangju Institute of Science and Technology, 123 Chumdangwagi-ro, Buk-gu, Gwangju 61005 South Korea

## Abstract

Quantum coherence control is reinvestigated for a new physical insight in quantum nonlinear optics and applied for a wavelength-convertible quantum memory in a solid ensemble whose spin states are inhomogeneously broadened. Unlike typical atomic media whose spin decays are homogeneous, a spin inhomogeneously broadened solid ensemble requires a counter-intuitive quantum coherence control to avoid spontaneous emission-caused quantum noises. Such a quantum coherence control in a solid ensemble satisfying both near perfect retrieval efficiency and ultralong photon storage offers a solid framework to quantum repeaters, scalable qubit generations, quantum cryptography, and highly sensitive magnetometry. Here, the basic physics of the counter-intuitive quantum coherence control is presented not only for a fundamental understanding of collective ensemble phase control but also for a coherence conversion mechanism between optical and spin states involving Raman rephasing.

## Introduction

Quantum coherence control in a lambda-type three-level optical ensemble has drawn much attention for various applications of quantum nonlinear optics over the last several decades, ranging from Kerr nonlinearity to quantum information, where a controlled coherence conversion (CCC) between optical and spin states plays a key role. Of them are electromagnetically induced transparency^1–3^, nondegenerate four-wave mixing (NDFWM)^4–11^, resonant Raman echoes^6^, ultraslow and stopped lights^7–10^, stationary lights^11,12^, Schrodinger’s cats^13,14^, entangled photon-pair generations^15–19^, quantum cryptography^20^, and photon echo-based quantum memories^21–33^. An essential requirement for the Raman-type nonlinear quantum optics is a long coherence time between two-ground (or spin) states^3^. In the study of photon echo-based quantum memories, CCC has been intensively studied recently to investigate the physical mechanism of collective phase manipulations of an ensemble^21–23,29,30^. The multimode storage capability is an intrinsic property of photon echoes and advantageous for mass information processing compared with single mode property in cavity quantum electrodynamics^34^, off-resonant Raman scattering^15–20^, single color center diamonds^35–37^, and even stopped lights^7–12^.

Since the first modified photon echo protocol was proposed for quantum memories in a spin homogeneous Doppler medium^21^, several methods have followed in the name of atomic frequency comb echoes^24,25^, gradient echoes^26^, and controlled double rephasing (CDR) echoes^22,23,29^ mostly in solid media. In quantum memories, both near perfect retrieval efficiency and ultralong storage time are the most important properties to be satisfied for recursive operations such as in circuit-based quantum computing^38^ and quantum repeaters for long-distance quantum communications^39^. The ultralong coherence time^40–42^ is also essential in magnetometry for ultrahigh sensitivity^43,44^. Although the CDR echo protocol satisfies both high retrieval efficiency and ultralong storage time, the optical pulse scheme is rather complex^22,23,29^. Moreover, CCC has never been discussed for a spin inhomogeneity in an optical ensemble, where most solid media are belong to this category. Although, CCC in a Doppler medium has opened a door to practical quantum optical memories with near perfect retrieval efficiency and longer storage time^21^, the same method cannot be applied to a non-Doppler solid ensemble^24^ simply because there is no π phase cancellation^6,22,23,29,30^, unless Raman rephasing is applied^9^, resulting in an absorptive coherence on the output photons. So far, there is no observation of quantum memory satisfying both near perfect retrieval efficiency and spin coherence-limited storage time, yet.

**Figure 1:**
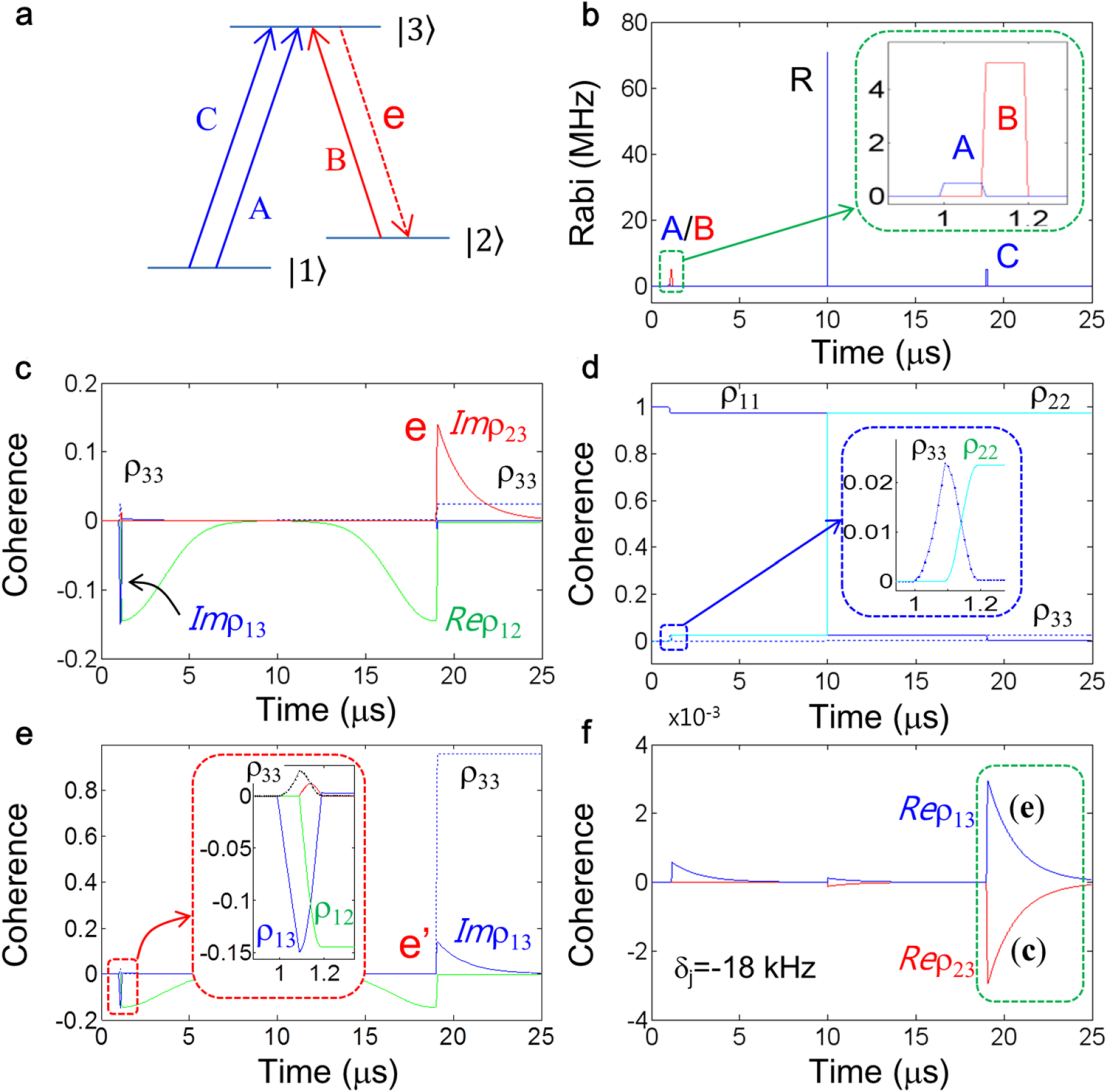
Controlled echo in a spin inhomogeneously broadened three-level optical ensemble. (**a**) A lambda-type energy level diagram interacting with optical pulses. (**b**) Pulse sequence of (**a**). Resonant Raman rephasing pulse R is composed of equal Rabi frequency of A and B. The control pulse C is resonant for the transition of either $|1\rangle -|3\rangle$ or $|2\rangle -|3\rangle$. The pulse arrival time of A, B, R, and C is ${t}_{A}=1.0\,{\rm{\mu }}s$, ${t}_{B}=1.1\,{\rm{\mu }}s$, ${t}_{R}=10.0\,{\rm{\mu }}s$, and ${t}_{C}=19.0\,{\rm{\mu }}s$, respectively. Each pulse duration is 0.1 μs except for R at 0.01 μs. (**c**) and (**d**) Coherence and population evolutions for (**a**) and (**b**). Blue: *Im*ρ_33_, Red: *Im*ρ_23_, Green: *Re*ρ_12_, Cyan: ρ_22_, Dotted: ρ_33_. (**e**) Numerical results when C is for $|2\rangle -|3\rangle$ transition: ρ_33_ ≫ ρ_11_ at t > t_e_, (19.1 μs). (**f**) Coherence evolutions of real components for two different access of C for (**c**) and (**e**) (overlapped). The detuning δ_j_ is for the j^th^ detuned spin. All decay rates are zero except for phase relaxation rates γ_31_ = γ_32_ = 50 Hz. The spin inhomogeneous width (FWHM) of $|1\rangle -|2\rangle$ transition is 170 kHz. The Rabi frequency of R is ${{\rm{\Omega }}}_{R}=100/\sqrt{2}$ MHz. The Rabi frequency Ω_A_, Ω_B_, and Ω_C_ is 0.5, 5, and 5 MHz, respectively. All numbers in decay rates and Rabi frequencies are divided by 2π.

**Figure 2:**
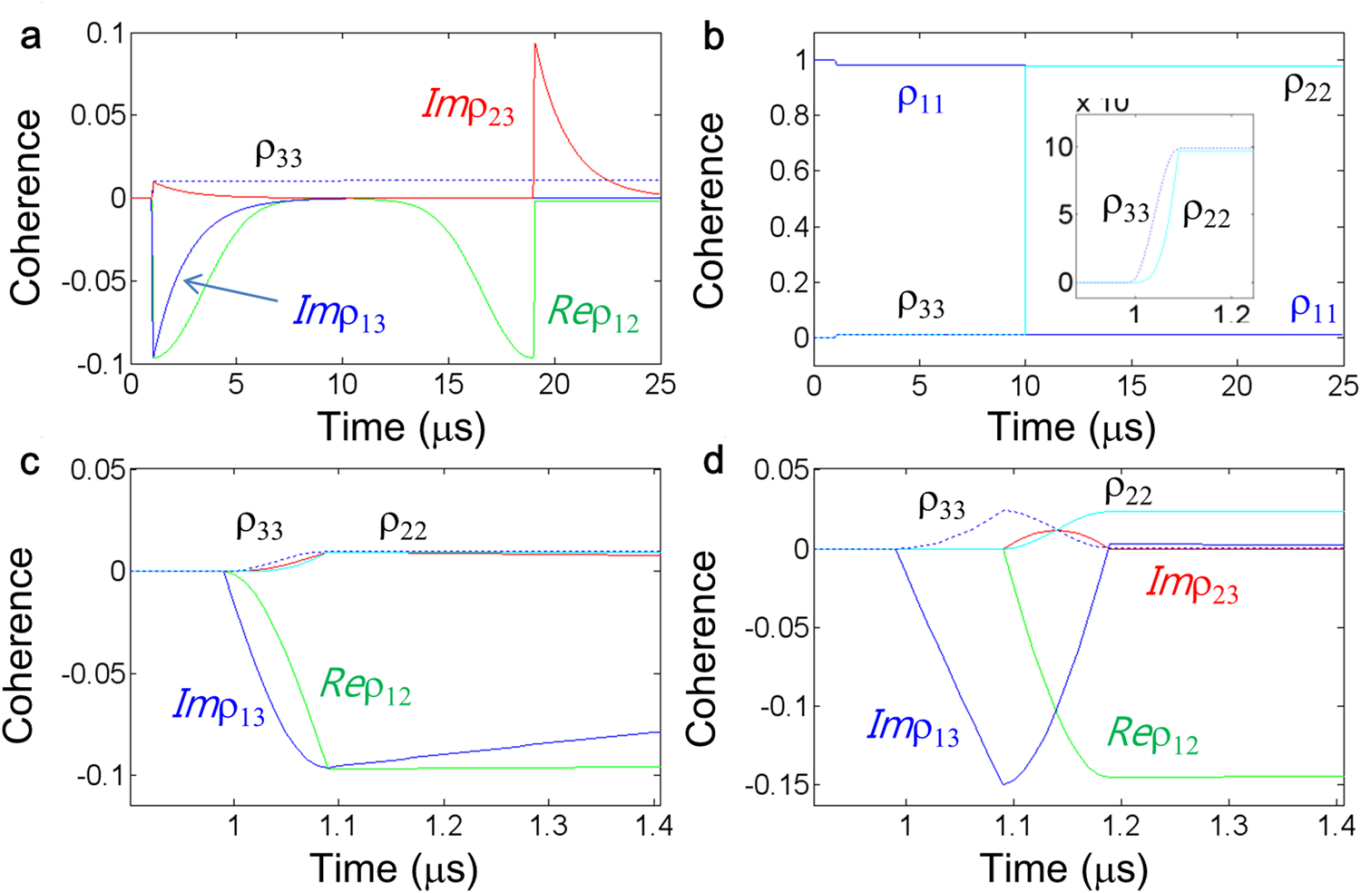
Controlled echo calculations for resonant Raman data. (**a**)-(**c**) Numerical results of Fig. 1(c) when A and B in Fig. 1 form a resonant Raman pulse. All others are same as Fig. 1. Dotted: ρ_33_; cyan: ρ_22_; Red: *Im*ρ_23_; Blue: *Im*ρ_13_; Green: *Re*ρ_12_. (**d**) Details of Fig. 1(c) to compare with (**c**). The pulse area of the data D is π, where the generalized Rabi frequency of D is ${{\rm{\Omega }}}_{D}=\sqrt{{{\rm{\Omega }}}_{A}^{2}+{{\rm{\Omega }}}_{B}^{2}}=5\,MHz$. Ω_A_ = 0.5 MHz. All other parameters are the same as in Fig. 1.

**Figure 3:**
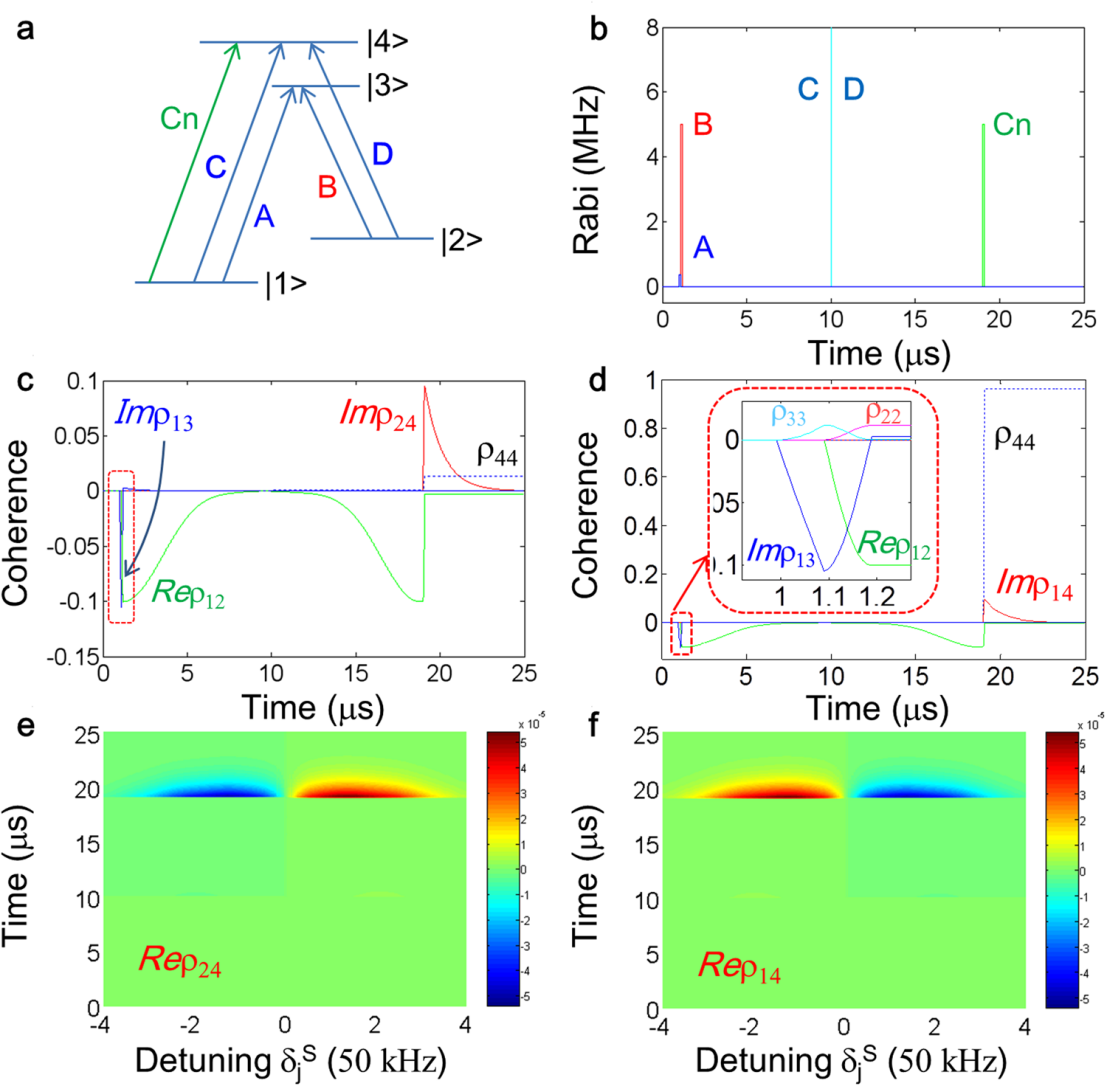
A wavelength-convertible controlled echo. (**a**) and (**b**) Energy level diagram and pulse sequence. The optical pulses C and D are for resonant Raman rephasing whose pulse area is 2π, and each Rabi frequency is ${{\rm{\Omega }}}_{C}={{\rm{\Omega }}}_{D}=100/\sqrt{2}\,{\rm{MHz}}$. The control Rabi frequency Cn is ${{\rm{\Omega }}}_{Cn}=5\,\mathrm{MHz}$. (**c**–**f**) Numerical calculations, where Cn is for the transition $|1\rangle -|4\rangle$ in (**c**) and (**e**), and $|2\rangle -|4\rangle$ in (**d**) and (**f**). The dotted box in (**c**) is the same as the inset in (**d**). All decay rates are zero except for the optical homogeneous decay rates of 150 kHz. All other parameters are the same as in Fig. 1.

Unlike vastly studied alkali atoms for quantum nonlinear optics^1,3,4,7,8,10,11,15–21,32,40,42^, solid media such as rare-earth doped crystals^2,5,6,9,13,14,22–31,41,45–49^ and color center diamonds^33,35–37,43,44^ have an intrinsic property of spin inhomogeneity. In general, the spin inhomogeneity deteriorates optical nonlinear efficiency due to coherence dephasing^9,24,30^, so that such a solid ensemble has been strictly prevented from time-delayed operations such as photon storage unless rephrasing is involved^9^. Here, the so-called *controlled echo* is proposed, analyzed, and discussed for CCC in a spin inhomogeneously broadened solid ensemble for the first time. The *controlled echo* is also presented for a *wavelength-convertible quantum memory* satisfying both ultralong storage time and near perfect retrieval efficiency, where its retrieval mechanism is new with a succinct pulse scheme. Surprisingly the theoretical investigation of the present *controlled echo*-based quantum memory reveals that our conventional understanding on quantum coherence control is actually a special case, and thus it has misled quantum optics community working on solid media so far^24,27,33^. For this, a simple lambda-type three-level optical system is first investigated to derive the basic physics of quantum coherence control for the *controlled echo* in Figs 1 and 2. Then, a *wavelength-convertible quantum memory* is presented in a double-lambda-type four-level system in Fig. 3.

Figure 1a shows the energy level diagram of a typical lambda-type three-level optical medium, but the access of the control pulse C is counter-intuitive for the present controlled echo-based quantum memory, where the data pulse A is to be stored. This counter-intuitive control access is the essence of the *controlled echo*, where its physics investigation is the key task of the present paper. Figure 1b is the pulse sequence for Fig. 1a, and the Raman rephasing pulse R is composed of a balanced Raman pulse for $|1\rangle -|3\rangle -|2\rangle$ transition for the control of spin coherent transients. The NDFWM signal **e** in Fig. 1c results from the light-matter interactions among optical pulses A, B, and C, where Raman rephasing by R play a key role. In spin homogeneous media such as alkali atoms^4,7,8,10,11,15–21,32,40,42^, however, the control pulse C must be the same as B, if they are collinear, for the transition $|2\rangle -|3\rangle$ without R^7,8,10,11,21^, resulting in NDFWM signal **e’** in the same frequency as A (see Fig. 1e).

To describe the basic physics of the *controlled echo*, first, analytic discussions are performed and then numerical demonstrations follow to prove them. According to the CDR theory^22,23,29^, the function of CCC is for the coherence conversion between optical and spin states by the control pulse B acting on the data pulse-excited population ρ_33_ on the excited state $|3\rangle$:

1 $${\rho }_{13}\mathop{\to }\limits^{B(\pi )}{\rho }_{12}\,(\,=\,-\,i\cdot {\rho }_{13}),$$

where ρ_33_ is completely transferred onto an auxiliary ground state $|2\rangle$ by B: ${\rho }_{33}\mathop{\longrightarrow }\limits^{B(\pi )}{\rho }_{22}$. Here, B(π) stands for a π pulse area of B. The successive CCC by the second control pulse C results in coherence inversion:

2 $${\rho }_{13}({t}_{C})=-\,{\rho }_{13}({t}_{A}),$$

where C is identical to B for the same transition. Equation (2) represents a correct understanding of conventional NDFWM process without rephrasing^7,8^. As a result, there is a π phase shift between the input and output^21–23,29,30^. Because the input pulse A must induce absorptive coherence ρ_13_(t_A_) via absorption process, the π-phase shift in equation (2) to the output represents emissive coherence. Thus, the output coherence either from a rephasing system applied by an identical control pulse set^24^ or from a doubly rephrased system^27,33^ must be also absorptive^29^ (see the Supplementary Information Fig. S1).

In a solid ensemble whose spin transition is inhomogeneous, however, the Raman rephasing by R is required as shown in Fig. 1b, otherwise the system is quickly dephased within spin T_2_^*^^9,24,30^. Here, I introduce new physics of *controlled echo* in Kerr nonlinear optics. By using equation (1) and the following Raman rephrasing by R [${\rho }_{21}({t}_{C})={\rho }_{12}^{\ast }({t}_{B})]$, the NDFWM signal **e** at t = t_e_ in Fig. 1a is expressed by:

3 $${\rm{Pulse}}\,{\rm{B}}(|2\rangle -|3\rangle ):{\rho }_{12}({t}_{B})=-\,i{\rho }_{13}({t}_{A}),$$

4 $${\rm{Pulse}}\,{\rm{C}}(|1\rangle -|3\rangle ):{\rho }_{23}({t}_{e})=-\,i{\rho }_{21}({t}_{C})=-\,{\rho }_{13}({t}_{A}),$$

where the control access of C must be counter-intuitive for the transition $|1\rangle -|3\rangle$ to work with the same transferred atom (ion) by B, satisfying no spontaneous emission-caused quantum noise. Here, equation (4) is the same as the conventional case in equation (2). Thus, the present *controlled echo* in Fig. 1 works for the quantum memory with emissive coherence under no population inversion via unitary transformation of Raman rephasing. By the way, if C is identical to B for the same transition^9^, the NDFWM signal **e’** at t = t_e_ becomes:

5 $${\rm{C}}(|2\rangle -|3\rangle ):{\rho }_{13}({t}_{e})=-\,i{\rho }_{12}({t}_{C})={\rho }_{13}^{\ast }({t}_{A}),$$

where ${\rho }_{33}({t}_{e})\gg {\rho }_{22}({t}_{e})$, causing spontaneous emission noise (see Fig. 1e).

The *controlled echo* in equation (4) is now discussed in more detail for the coherent transients. By the first control pulse B, the transferred coherence ρ_12_ in equation (3) begins to evolve due to the spin inhomogeneity:

6 $${{\rm{\rho }}}_{12}({\rm{t}})=-\,i{{\rm{\rho }}}_{13}({t}_{A}){e}^{\pm i{\delta }_{j}t}:{t}_{B}\le t < {t}_{R},$$

where ${\delta }_{j}$ is the detuning of the j^th^ spin from the two-photon line center, and *t*_*k*_ is the ending time of pulse *k*. Here, the pulses A and B must be consecutive to neglect optical dephasing regardless of the optical inhomogeneity. By the Raman rephasing pulse R at t = t_R_, the completely dephased spin coherence ρ_12_(t_R_) starts to rephase:

7 $${{\rm{\rho }}}_{12}({\rm{t}}^{\prime} )=i{\rho }_{13}^{\ast }({t}_{A}){e}^{\pm i{\delta }_{j}(t^{\prime} -T)}:{t}_{R}\le t^{\prime}  < {t}_{C},$$

where ${\rm{T}}\equiv ({t}_{R}-{t}_{B})=({t}_{C}-{t}_{R})$ and $t^{\prime} =(t-{t}_{B})-T$. Therefore, at ${\rm{t}}={t}_{B}+2{\rm{T}}$, the rephrased spin coherence reaches at maximum value (spin echo) with a π/2 phase shift:

8 $${{\rm{\rho }}}_{12}({t}_{C^{\prime} })=i{\rho }_{13}^{\ast }({t}_{A}),$$

where ${t}_{C^{\prime} }$ is the arrival time of the pulse C. If the second control pulse C follows the conventional NDFWM process as in equation (2), the output **e’** becomes:

9 $${\rho }_{13}(t^{\prime\prime} )=-\,i{\rho }_{12}({t}_{C}){e}^{-\frac{{t}^{^{\prime\prime} }}{{T}_{2}}}={\rho }_{13}^{\ast }({t}_{A}){e}^{-\frac{{t}^{^{\prime\prime} }}{{T}_{2}}}:t^{\prime\prime} \ge {t}_{C}$$

where $\,t^{\prime\prime} =t-{t}_{C}$. The term ${e}^{-\frac{{t}^{^{\prime\prime} }}{{T}_{2}}}$ is due to optical dephasing, where T_2_ is replaced by the laser jitter-induced ${T}_{2}^{\ast }(\,\ll \,{T}_{2})$ in rare earth doped solids^2,5,6,9,24–26,30^. In equation (9), the output **e’** is obviously violates quantum memory due to population inversion (${\rho }_{33}\gg {\rho }_{11}$). If there is no Raman rephasing as in ref.^24^, then, the output **e’** still becomes absorptive due to the intrinsic optical rephrasing property^25,31^, which again violates the quantum memory (will be discussed elsewhere).

On the contrary, for the case of *controlled echo* in Fig. 1a, the final coherence for the NDFWM signal **e** becomes:

10 $${\rho }_{23}(t^{\prime\prime} )=-\,i{\rho }_{21}({t}_{C}){e}^{-\frac{{t}^{^{\prime\prime} }}{{T}_{2}}}=-\,{\rho }_{13}({t}_{A}){e}^{-{t}^{^{\prime\prime} }/{T}_{2}},$$

where ${{\rm{\rho }}}_{21}={\rho }_{12}^{\ast }$ and ${\rho }_{33}({t}_{C})={\rho }_{33}({t}_{A})$. Thus, the NDFWM signal **e** at t = t_e_ is described as:

11 $${\rho }_{23}({t}_{e})=-\,{\rho }_{13}({t}_{A}),$$

where ${\rho }_{33}({t}_{e})\ll {\rho }_{22}({t}_{e})$ due to the Raman rephrasing by R at t = t_R_. In equations (9) and (10), both NDFWM signals, **e’** and **e**, represent the same emissive coherence, because all real components are zero at t = t_e_ (see the Supplemental Information Fig. S2). Thus, the present *controlled echo* in Fig. 1a is now analytically proved for a quantum memory protocol in a spin inhomogeneously broadened solid ensemble.

Figure 1c–f represent numerical results for the *controlled echo* in Fig. 1a: see *Methods* for details. Figure 1c is the numerical result of the *controlled echo*, where the control pulse access is counter-intuitive with respect to conventional one (see Fig. 1e). In Fig. 1c, the data pulse-excited coherence ρ_13_(t_A_) is transferred into spin coherence ρ_12_(t_B_) by the first control pulse B as discussed in equation (6) (see the inset in Fig. 1e). The maximally rephased coherence (ρ_12_^*^) by R is again coherently transferred by the second control pulse C, resulting in the emissive NDFWM signal **e** [*Im*ρ_23_(t_e_)] (see the red curve) under no population inversion, as discussed in equation (11).

The magnitude of **e** $(Im{\rho }_{23}\,(t\ge {t}_{e}))$ in Fig. 1c should decay down exponentially as a function of time by optical phase relaxation rate γ_23_ (1/T_2_). This decay is of course accelerated by optical inhomogeneous broadening. The excited state population ρ_33_ (see the dotted line) for **e** at t = t_e_ is exactly the same as that for A at t = t_A_ (see the dotted line and the inset in Fig. 1d), resulting in no spontaneous emission-caused quantum noise. Figure 1d shows population evolutions for Fig. 1c, where the data A-excited population ρ_33_ (dotted curve/line) is fully transferred into state $|2\rangle$ by B at t = 1.2. The function of R at t = t_R_ is to swap the population between two ground states $|1\rangle$ and $|2\rangle$, resulting in spin rephasing^5,6,9,48^: ${\rho }_{11}\mathop{\longleftrightarrow }\limits^{R(2\pi )}{\rho }_{22}$ and $\,{\rho }_{12}\mathop{\longrightarrow }\limits^{R(2\pi )}{\rho }_{12}^{\ast }$; For the physics of 2π pulse area of R, refer to refs^6,48^.

In contrast to Fig. 1c, Fig. 1e is for the conventional control pulse access to the transition $|2\rangle -|3\rangle$, resulting in an emissive **e’** output (*Im*ρ_13_) at the same frequency as A for the transition $|1\rangle -|3\rangle$^7–10^. The emissive NDFWM signal **e’** at ${\rm{t}}\ge {t}_{e}$ is, however, under population inversion as shown in Fig. 1e: ρ_33_ ≫ ρ_11_. Figure 1f shows the relation between equations (9) and (11). Thus, the present *controlled echo* theory is numerically proved for quantum memories.

Figure 2 represents numerical calculations for the case of resonant Raman data pulse D, where the optical pulses A and B in Fig. 1b are simultaneous^5,6,48^. The pulse area of A is kept the same as Fig. 1 for the consistency. For maximum coherence excitation, the Raman data pulse area should be Φ_D_ = π, where ${{\rm{\Omega }}}_{D}=\sqrt{{{\rm{\Omega }}}_{A}^{2}+{{\rm{\Omega }}}_{B}^{2}}$ and ${{\rm{\Omega }}}_{A}\ll {{\rm{\Omega }}}_{B}$. Except for the Raman data D, all others are the same as in Fig. 1b. As shown in Fig. 2a, the *controlled echo* still works for the resonant Raman data for quantum memories. However, the excited population by the data pulse A is half-shelved in the excited state $|3\rangle$, where ρ_22_ = ρ_33_ (see Fig. 2b). Because the coherence is induced by the population difference, e.g., ${\rho }_{12}\propto {\rho }_{22}-{\rho }_{11}$, the spin coherence reduction in the resonant Raman case is ~67% (see Fig. 2c and d). Although the reduced coherence itself by the atom shelving has nothing to with quantum fidelity, the shelved population on $|3\rangle$ may deteriorate the read-out conversion efficiency by C. To avoid this matter, a double lambda-type four-level system may be used as in Fig. 3a, so that C pulse can circumvent the shelved population. The optical locking technique also gives a solution to avoid the shelving problem even for ultralong quantum memories^48^. In ref.^48^, quantum decoherence by spontaneous emission decay is negligible due to ${T}_{1}^{spin}\gg {T}_{1}^{optical}$, where the photon storage time is extended up to spin population decay time.

The Raman gradient-echo protocol based on off-resonant interactions may be free from the population shelving or population decay-caused quantum noises discussed in Fig. 2^32^. Instead of the direct Raman rephasing, an oppositely polarized gradient electric field applied to the C transition functions the Raman rephrasing without population control^32^. For the case of multiple data pulses (As), however, the mandatory use of a long, single control pulse C induces at least 50% coherence loss due to unwanted coherence read-out for ‘OFF’ data pulse timing. Moreover, such a Raman gradient technique can never be applied to a solid ensemble due to the intrinsic spin inhomogeneity.

Figure 3 is an application of the present *controlled echo* for a *wavelength-convertible quantum memory* in a double lambda-type four-level solid medium whose spin transition is inhomogeneouly broadened. For this, an extra state $|4\rangle$ is simply added in Fig. 1a. Unlike Fig. 1, the resonant Raman rephasing pulse R composed of balanced C and D is applied for the transition $|1\rangle -|4\rangle -|2\rangle$, so that any potential defect by the shelved atoms on state $|3\rangle$ can be removed on the coherence recovery process. The frequency difference between A and **e** is the origin of the frequency up- or down-conversion. The control pulse (Cn) access is for the transition $|1\rangle -|4\rangle$ and results in the photon signal **e**, resonant between states $|2\rangle$ and $|4\rangle$. Here it should be noted that the control pulse Cn must be resonant to maximize the coherence conversion process as shown in most quantum memory cases^7–10,15–23,28–30^, where preliminary NDFWM generation in a double-Λ solid medium of Fig. 3 has been observed in a cw scheme^49^. The Rabi frequency Ω_A_ of the data pulse A in Fig. 3 is decreased by a factor of $\sqrt{2}$ for the purpose of comparison with Fig. 1. As a direct result, the coherence excitation by A is also decreased by $\sqrt{2}$ as shown in Fig. 3c (see also the inset in Fig. 3d). Either for $|3\rangle$ or $|4\rangle$, a resonant Raman pulse deals only with two-photon coherence between the two ground states $|1\rangle$ and $|2\rangle$, resulting in the same spin rephasing result. The retrieved photon signal **e** must satisfy Kerr nonlinear optics among pulses A, B, and Cn, resulting in up- or down-conversion depending on the relative energy level of $|2\rangle$.

In contrast to Fig. 3c, the control pulse (Cn) access is applied for the transition $|2\rangle -|4\rangle$ in Fig. 3d, which is conventional as in Fig. 1e. The resultant coherence of the NDFWM signal **e’** is, however, under population inversion (ρ_44_ ~ 1 at t = t_e′_). Even if such an inverted echo signal may be useful for classical applications of associative memories^50^, the potential quantum noise from ρ_44_ should prevent Fig. 3d from quantum memories. The relation between equations (9) and (11) is also numerically demonstrated in Fig. 3e and f for the signals **e’** and **e** in Figs. 3c and d, respectively. This proves again a clear distinction between rephasing-based quantum memories and the present *controlled echo*: ρ^*^ vs. −ρ. Thus, the present *wavelength-convertible quantum memory* protocol in a resonant four-level solid system can be used for multimode quantum wavelength conversion, which is essential for future spectral division multiplexing in quantum networks.

For the near perfect retrieval efficiency in the present *controlled echo* quantum memory, the control pulse propagation directions must be opposite each other^21,30^, so that a backward echo signal **e** can be generated by the following phase matching conditions:

12 $${\omega }_{e}=-\,{\omega }_{A}+{\omega }_{B}+{\omega }_{C(Cn)},$$

13 $${{\boldsymbol{k}}}_{e}=-\,{{\boldsymbol{k}}}_{A}+{{\boldsymbol{k}}}_{B}+{{\boldsymbol{k}}}_{C(Cn)},$$

where ${\omega }_{j}$ (***k***_*j*_) is the angular frequency (wave vector) of pulse *j*, and the subscript ‘e’ stands for the *controlled echo*. Although a perfect collinear scheme between A and **e** cannot be satisfied due to ${{\boldsymbol{k}}}_{B}+{{\boldsymbol{k}}}_{Cn}\ne 0$^30^, the wavelength deviation among them is negligibly small at 10^−8^ for most rare-earth doped solids^46^. Thus, the refractive index-dependent phase walk-off is also negligibly small at far less than π as experimentally demonstrated^5,6,9,30^. This flexible NDFWM offers a great advantage in echo-based quantum memories for spatial multiplexing. According to the theory^21^ and experimental observations^30^, a near perfect retrieval efficiency η_e_ can be achieved for the backward controlled echo scheme even for an optically thick ensemble^45^: ${\eta }_{e}={(1-{e}^{-\alpha l})}^{2}$. Here, the higher optical depth (*αl*) is actually necessary for the single write- and read-out process, otherwise an optical cavity is needed sacrificing bandwidth. The above phase matching conditions can also be expanded for light polarizations.

In conclusion, a *wavelength-convertible quantum memory* protocol based on *controlled echo* was introduced, analyzed, and discussed for a spin inhomongeneously broadened double lambda-type four-level optical ensemble. Unlike alkali atoms whose spin transitions are homogeneous, the control pulse access in a spin inhomogeneous solid ensemble must be counter-intuitive to avoid spontaneous emission-caused quantum noises. In the present study, quantum coherence control in a spin inhomogeneously broadened solid ensemble was explicitly analyzed and discussed to elucidate the basic but novel physics of ensemble phase control for both quantum coherence conversion and Raman rephasing, resulting in near perfect, ultralong, and emissive photon echoes without quantum noises. With a backward control pulse set and balanced Raman rephasing, the retrieval efficiency can be near perfect due to the absence of echo reabsorption, and the photon storage time can be extended up to the spin homogeneous decay time. Here, the spin homogeneous decay time can be as long as minutes or even hours in rare-earth doped solids under external magnetic fields^41^. Moreover, the present *controlled echo* scheme is simple in configuration and applicable to spectral and spatial multiplexing in all-optical quantum information processing in the future quantum networks. In magnetometry, sensing ability increases by a factor of T_2_/T_2_^*^ ^44^, where T_2_ can be extended by several orders of magnitude in the present *controlled echo*. The present research sheds light on potential quantum memory applications in various quantum information areas such as scalable qubit generations, recursive operations, sensing, and quantum repeaters for long-distance quantum communications.

## Methods

For the numerical calculations, total sixteen time-dependent density matrix equations are solved for a four-level ensemble medium in an interacting Heisenberg picture under rotating wave approximations^51^: $\frac{d\rho }{dt}=\frac{i}{\hslash }[H,\rho ]-\frac{1}{2}\{{\rm{\gamma }},\rho \}$, where ρ is a density matrix element, H is Hamiltonian, and γ is a decay parameter. In the calculations, 99.55% of the Gaussian distribution is taken for total 201 distributed spin groups at 2 kHz spacing, where the spectral spin inhomogeneous width (FWHM) is set at 170 kHz. Here, the exaggerated spin bandwidth (x30) is only to save computer calculation time. Those 201 spectral groups are calculated for the time domain and summed up for all spectral groups. For the optical transition, optical inhomogeneity is neglected because it does not violate physics of Raman coherence nor affect the result, unless optical pulse delay between A and B is given. The following equations are for the coherence terms of ${\dot{\rho }}_{ij}$ in a four-level system interacting with three resonant optical fields:

14.1 $$\frac{d{\rho }_{12}}{dt}=\frac{i}{2}[{{\rm{\Omega }}}_{1}{\rho }_{32}-{{\rm{\Omega }}}_{2}{\rho }_{13}+{{\rm{\Omega }}}_{3}{\rho }_{42}-{{\rm{\Omega }}}_{4}{\rho }_{14}]-i{\delta }_{12}{\rho }_{12}-i({\delta }_{2}-{\delta }_{1}){\rho }_{12},$$

14.2 $$\frac{d{\rho }_{13}}{dt}=\frac{i}{2}[{{\rm{\Omega }}}_{1}({\rho }_{33}-{\rho }_{11})-{{\rm{\Omega }}}_{2}{\rho }_{12}]-i{\delta }_{1}{\rho }_{13}-{\gamma }_{13}{\rho }_{13},$$

14.3 $$\frac{d{\rho }_{14}}{dt}=\frac{i}{2}[{{\rm{\Omega }}}_{3}({\rho }_{44}-{\rho }_{11})-{{\rm{\Omega }}}_{4}{\rho }_{12}]-i{\delta }_{3}{\rho }_{14}-{\gamma }_{14}{\rho }_{14},$$

14.4 $$\frac{d{\rho }_{23}}{dt}=\frac{i}{2}[{{\rm{\Omega }}}_{2}({\rho }_{33}-{\rho }_{22})-{{\rm{\Omega }}}_{1}{\rho }_{21}]-i{\delta }_{2}{\rho }_{23}-{\gamma }_{23}{\rho }_{23},$$

14.5 $$\frac{d{\rho }_{24}}{dt}=\frac{i}{2}[{{\rm{\Omega }}}_{4}({\rho }_{44}-{\rho }_{22})-{{\rm{\Omega }}}_{3}{\rho }_{21}]-i{\delta }_{4}{\rho }_{24}-{\gamma }_{24}{\rho }_{24},$$

14.6 $$\frac{d{\rho }_{44}}{dt}=\frac{i}{2}[{{\rm{\Omega }}}_{3}({\rho }_{14}-{\rho }_{41})+{{\rm{\Omega }}}_{4}({\rho }_{24}-{\rho }_{42})]-({{\rm{\Gamma }}}_{41}+{{\rm{\Gamma }}}_{42}){\rho }_{44},$$

where the interaction Hamiltonian matrix H is given by:

15 $${\rm{H}}=-\,\frac{\hslash }{2}[\begin{array}{llll}-2{\delta }_{1} & 0 & {{\rm{\Omega }}}_{1} & {{\rm{\Omega }}}_{3}\\ 0 & -2{\delta }_{2} & {{\rm{\Omega }}}_{2} & 0\\ {{\rm{\Omega }}}_{1} & {{\rm{\Omega }}}_{2} & -2{\delta }_{3} & 0\\ {{\rm{\Omega }}}_{3} & 0 & 0 & 0\end{array}].$$

here Ω_1_ (Ω_3_) is the Rabi frequency of the optical field between the ground state $|1\rangle$ and the excited state $|3\rangle$ ($|4\rangle$), and Ω_2_ (Ω_4_) is the Rabi frequency of the optical field between the ground state $|2\rangle$ and the excited state $|3\rangle$ ($|4\rangle$). The δ_1_, δ_2_, and δ_3_ are the atom detuning from the resonance frequency for Ω_1_, Ω_2_, and Ω_3_ (Ω_4_), respectively. For visualization purpose and simplification, all decay terms are neglected except for the optical phase relaxation rates γ_ij_.

The optical pulse duration is set at 0.1 μs, otherwise specified. The time increment in the calculations is 0.01 μs. Initially all atoms are in the ground state $|1\rangle$ (${\rho }_{11}(0)=1$), and thus all initial coherence is ${\rho }_{ij}(0)=0$, where *i* (*j*) = 1, 2, 3, 4. The program used for the numerical calculations is time-interval independent, so that there is no accumulated error depending on the time interval settings. The corresponding Rabi frequency is set at $\frac{100}{\sqrt{2}}$ MHz for the pulse duration of 0.01 μs to satisfy a 2π pulse area.

## Electronic supplementary material

Supplementary information

## References

[1] Harris SE (1997). Electromagnetically induced transparency. Phys. Today.

[2] Ham BS, Hemmer PR, Shahriar MS (1997). Efficient electromagnetically induced transparency in a rare-earth doped crystal. Opt. Commun..

[3] Fleischhauer M, Lukin MD (2000). Dark-state polaritons in electromagnetically induced transparency. Phys. Rev. Lett..

[4] Li YQ, Xiao M (1996). Enhancement of nondegenerate four-wave mixing based on electromagnetically induced transparency in rubidium atoms. Opt. Lett..

[5] Ham BS, Shahriar MS, Hemmer PR (1997). Enhanced nondegenerate four-wave mixing owing to electromagnetically induced transparency in a spectral hole-burning crystal. Opt. Lett..

[6] Ham BS, Shahriar MS, Kim MK, Hemmer PR (1998). Spin coherence excitation and rephasing with optically shelved atoms. Phys. Rev. B.

[7] Liu C, Dutton Z, Behroozi CH, Hau LV (2001). Observation of coherent optical information storage in an atomic medium using halted light pulses. Nature.

[8] Choi KS, Deng H, Laurat J, Kimble HJ (2008). Mapping photonic entanglement into and out of quantum memory. Nature.

[9] Turukhin AV (2002). Observation of ultraslow and stored light pulses in a solid. Phys. Rev. Lett..

[10] Hsiao Y-F (2018). Highly efficient coherent optical memory based on electromagnetically induced transparency. Phys. Rev. Lett..

[11] Moiseev SA, Ham BS (2006). Quantum manipulation of two-color stationary light: Quantum wavelength conversion. Phys. Rev. A.

[12] Everett JL (2017). Dynamical observations of self-stabilizing stationary light. Nature Phys..

[13] Petrosyan D, Kurizki G (2002). Symmetric photon-photon coupling by atoms with Zeeman-split sublevels. Phys. Rev. A.

[14] Peternostro M, Kim MS, Ham BS (2003). Generation of entangled coherent states via cross-phase-modulation in a double electromagnetically induced transparency regime. Phys. Rev. A.

[15] van der Wal CH (2003). Atomic memory for correlated photon states. Science.

[16] Balic´ V, Braje DA, Kolchin P, Yin GY, Harris SE (2005). Generation of Paired Photons with Controllable Waveforms. Phys. Rev. Lett..

[17] Julsgaard B, Sherson J, Cirac JI, Fiurášek J, Polzik ES (2004). Experimental demonstration of quantum memory for light. Nature.

[18] Ding D-S (2015). Raman quantum memory of photonic polarized entanglement. Nature Photon..

[19] Parniak M (2017). Wavevector multiplexed atomic quantum memory via spatially-resolved single-photon detection. Nature Communi..

[20] Zhang W (2017). Quantum secure direct communication with quantum memory. Phys. Rev. Lett..

[21] Moiseev SA, Kröll S (2001). Complete reconstruction of the quantum state of a single-photon wave packet absorbed by a Doppler-broadened transition. Phys. Rev. Lett..

[22] Ham, B. S. Coherent control of collective atom phase for ultralong, inversion-free photon echoes. *Phys*. *Rev*. A **8**5, 031402(R) (2012); *ibi*d, *Phys*. *Rev*. A **9**4, 049905(E) (2016).

[23] Ham BS (2017). A controlled ac Stark echo for quantum memories. Sci. Rep..

[24] Afzelius M (2010). Demonstration of atomic frequency comb memory for light with spin-wave storage. Phys. Rev. Lett..

[25] de Riedmatten H, Afzelius M, Staudt MU, Simon C, Gisin N (2008). A solid-state light–matter interface at the single-photon level. Nature.

[26] Hetet G, Longdell JJ, Alexander AL, Lam PK, Sellars MJ (2008). Electro-Optic Quantum Memory for Light Using Two-Level Atoms. Phys. Rev. Lett..

[27] Damon V, Bonarota M, Louchet-Chauvet A, Chanelière T, Le Gouët J-L (2011). Revival of silenced echo and quantum memory for light. New. J. Phys..

[28] Lvovsky AI, Sanders BC, Tittlel W (2009). Optical quantum memory. Nature Photon..

[29] Ham BS (2018). Control Rabi flopping applied to photon echoes for quantum memories. Adv. Appl. Sci. Res..

[30] Hahn J, Ham BS (2011). Rephasing halted photon echoes using controlled optical deshelving. New J. Phys..

[31] Usmani I, Afzelius M, de Riedmatten H, Gisin N (2010). Mapping multiple photonics qubits into and out of one solid-state atomic ensemble. Nature Communi..

[32] Hosseini M, Sparkes BM, Campbell G, Lam PK, Buchler BC (2011). High efficiency coherent optical memory with warm rubidium vapour. Nature Communi..

[33] Julsgaard B, Grezes C, Bertet P, Mølmer K (2013). Quantum memory for microwave photons in an inhomogeneously broadened spin ensemble. Phys. Rev. Lett..

[34] Maıˆtre X (1997). Quantum Memory with a Single Photon in a Cavity. Phys. Rev. Lett..

[35] Togan E (2010). Quantum entanglement between an optical photon and a solid-state spin qubit. Nature.

[36] Fuchs GD, Burkard G, Klimov PV, Awschalom DD (2011). A quantum memory intrinsic to single nitrogen-vacancy centers in diamond. Nature Phys..

[37] England DG (2015). Storage and retrieval of THz-bandwidth single photons using a room-temperature diamond quantum memory. Phys. Rev. Lett..

[38] Steane AM (1999). Efficient fault-tolerant quantum computing. Nature.

[39] Duan L-M, Lukin MD, Cirac JI, Zoller P (2001). Long-distance quantum communication with atomic ensembles and linear optics. Nature.

[40] Langer C (2005). Long-Lived Qubit Memory Using Atomic Ions. Phys. Rev. Lett..

[41] Zhong M (2015). Optically addressable nuclear spins in a solid with a six-hour coherence time. Nature.

[42] Bao X-H (2012). Efficient and long-lived quantum memory with cold atoms inside a ring cavity. Nature Phys..

[43] Zaiser S (2016). Enhancing quantum sensing sensitivity by a quantum memory. Nature Communi..

[44] Taylor JM (2008). High-sensitivity diamond magnetometer with nanoscale resolution. Nature Phys..

[45] Sangouard N, Simon C, Afzelius M, Gisin N (2007). Analysis of a quantum memory for photons based on controlled reversible inhomogeneous broadening. Phys. Rev. A.

[46] MacFarlane, R. M. & Shelby, R. M. *Coherent Transients and Holeburning Spectroscopy of Rare Earth Solids*, Spectroscopy of Solids Containing Rare Earth Ions, edited by Kaplyanskii, A. A. & MacFarlane, R. M., Chap. 3. (Elsevier Science Publishers, New York, 1987).

[47] Mitsunaga M (1990). cw photon echo: Theory and observation. Phys. Rev. A.

[48] Ham BS (2009). Ultralong quantum optical data storage using an optical locking technique. Nature Photon..

[49] Ham BS, Shahriar MS, Hemmer PR (1999). Enhancement of four-wave mixing and line narrowing by use of quantum coherence in an optically dense double-lambda solid. Opt. Lett..

[50] Kohonen, T. *Self-organization and associative memory*, 3^rd^ ed., Springer-Verlag (1987).

[51] Sargent, M. III, Scully, M. O. & Lamb, W. E. Jr., *Laser Physics*, Addison-Wesley (1974).

