# Supplementary material for: A wavelength-convertible quantum memory: Controlled echo
Source: Sci Rep. 2018 Jul 16;8:10675. doi: 10.1038/s41598-018-28776-1 (PMC6048175; doi:10.1038/s41598-018-28776-1)
Supplement: Supplementary file 1 — Supplementary information [file 41598_2018_28776_MOESM1_ESM.pdf]

Supplementary Information for

A wavelength convertible quantum memory: Controlled echo

Byoung S. Ham
GIST, S. Korea
bham@gist.ac.kr

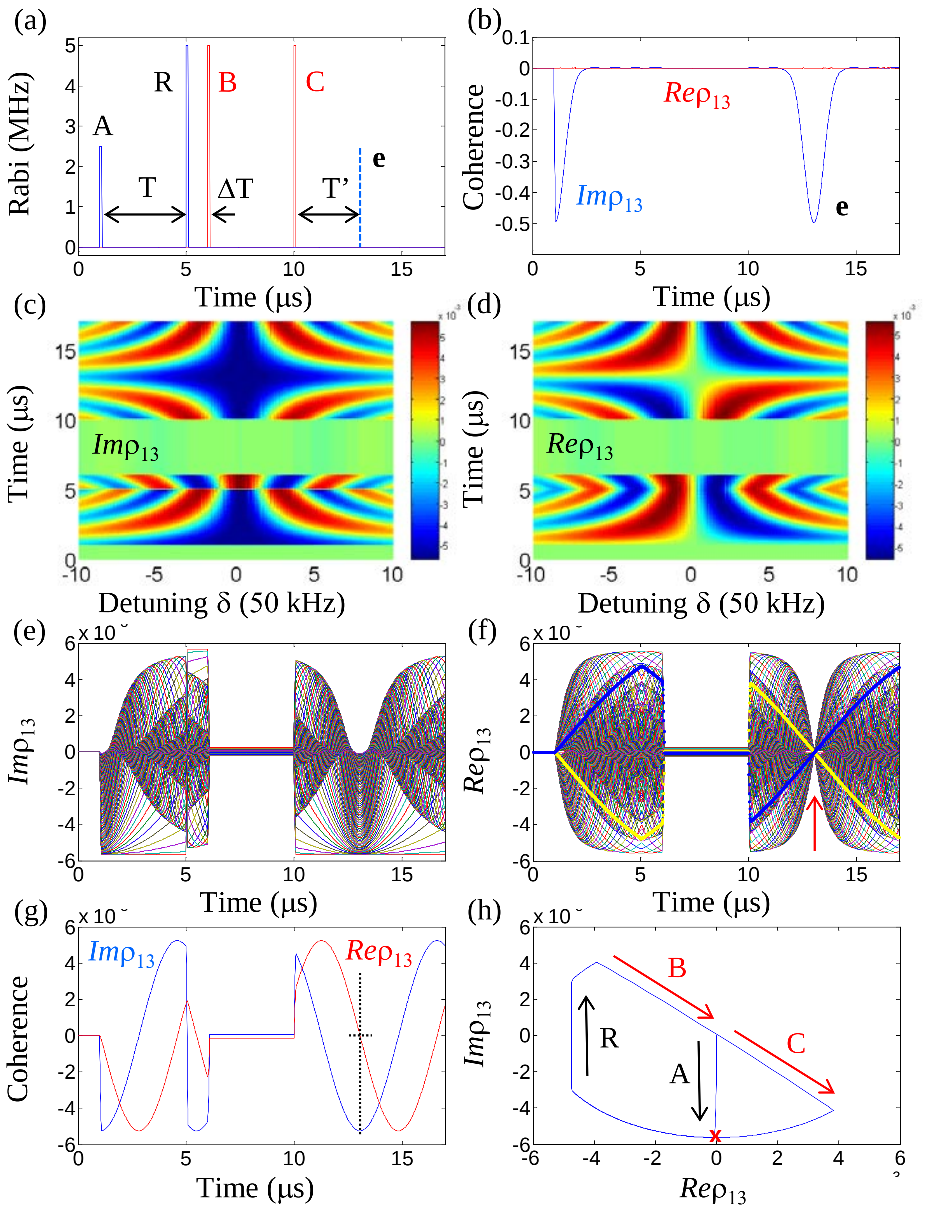

Fig. S1. Controlled coherence conversion applied to a two-pulse photon echo in a three-level system. (a) Pulse sequence. (b)-(h) Numerical calculations for (a). The control pulse area of B and C is π. No spin inhomogeneity is assumed. The blue (yellow) curve is for symmetrically detuned atom pair at $\pm\delta_j$. The optical inhomogeneous width is Δ_{inh}=510 kHz (FWHM; Gaussian). The pulse arrival time is t_A=1 μs, t_R=5 μs, t_B=6 μs and t_C=10 μs. ΔT=1 μs.

Figure S1 shows quantum coherence control applied to conventional photon echoes. The control pulses B and C are identical with a π pulse area and resonant between states $|2\rangle$ and $|3\rangle$. The pulses A and R are resonant between $|1\rangle$ and $|3\rangle$, whose pulse are is π/2 and π, respectively. The pulse delay between R and B is intentionally given by ΔT to check the effect of quantum coherence control with respective to photon rephasing. The spin inhomogeneity (Δ_s) affects overall decoherence as a function of e^{-t/T_2^*}, where $T_2^* = 1/\pi\Delta_S$.

Figure S1(b) represents an absorptive photon echo resulting from the quantum coherence control. The absorptive echo is due to coherence inversion by the control Rabi flopping by B(π) and C(π). As shown in Figs. S1(c)-(h), the control pulses do not affect the photon echo except for the delay determined by $(t_C - t_B)$, and

the coherence inversion: $t_e = t_C + T - \Delta T$=13 μs; $T' = T - \Delta T$. Although quantum coherence control results in the same effect onto the photon echo due to symmetric distribution of the real components ($Re\rho_{13}$); see the blue and yellow curves for $\pm\delta_j$ in Fig. S5(f)), the physics is completely different as shown in Fig. S1(h). Thus, the controlled atomic frequency comb (AFC) echo [20] also results in absorptive coherence, because AFC echo is based on accumulated echo, where the read pulse is replaced by a single photon data in AFC [43]. The same absorptive coherence phenomenon on the echo in the double rephrasing case is shown in Figs. S2(i) and (j).

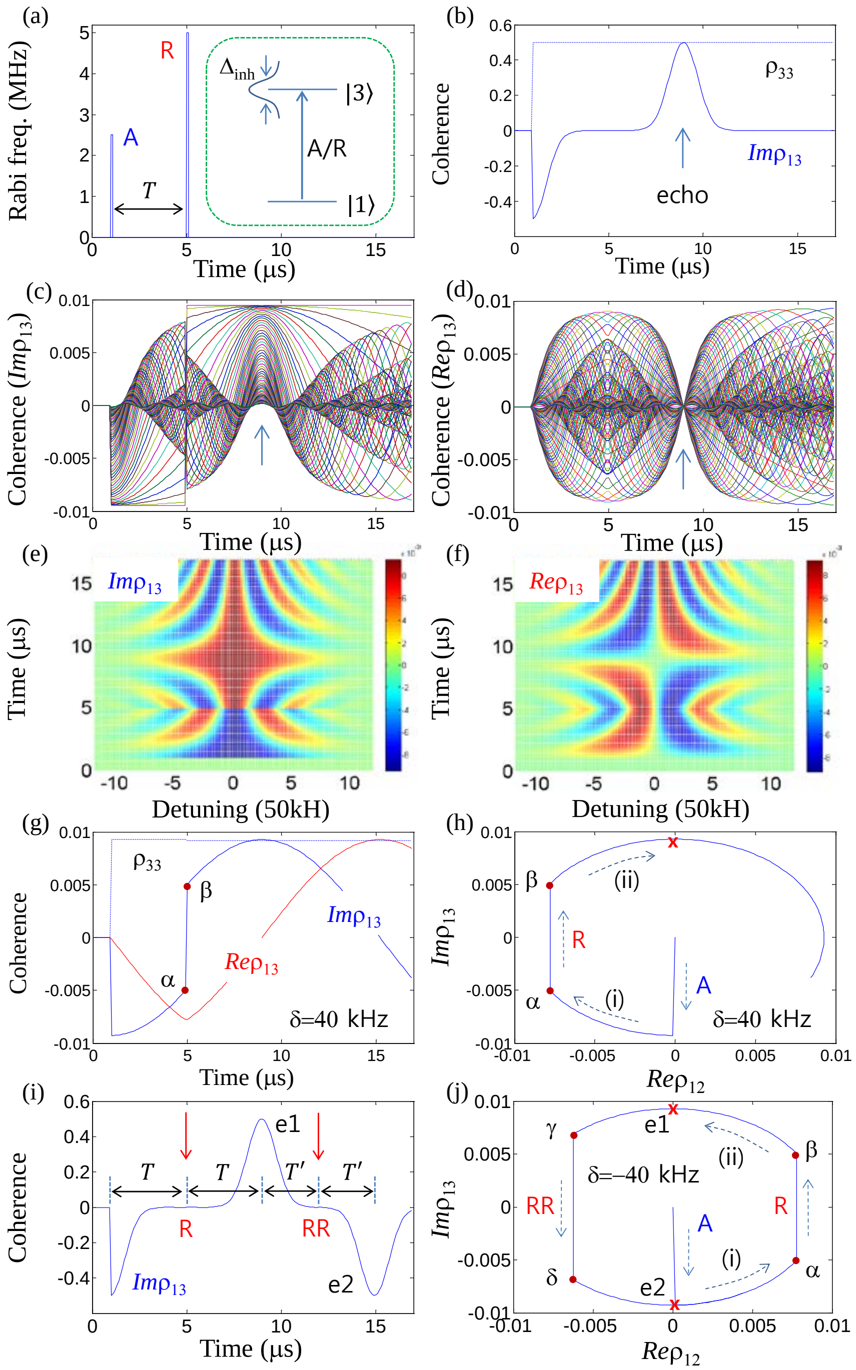

Fig. S2. A two-pulse photon echo in a two-level system. (a) Pulse sequence. The pulse area of D and R is $\pi/2$ and π, respectively. (b)-(h) Numerical calculations for (a). (b) Overall coherence evolution. (c) and (d) Individual coherence evolutions in (b). (e) and (f) 3D plots for (c) and (d), respectively. (g) Symmetric ($Re\rho_{13}$) and anti-symmetric ($Im\rho_{13}$) features across the rephasing pulse R. (h) a Bloch vector model for an individual coherence evolution for (g). (i) and (j) A double rephasing echo with an added second rephasing pulse RR at t=12 μs. The asymmetric coherence inversion between α and β (γ and δ) stands for rephasing by R (RR).The pulse arrival time is t_A=1 μs; t_R=5 μs; and t_{RR}=12 μs. The optical inhomogeneous width is Δ_{inh}=510 kHz (FWHM; Gaussian). The e1 (e2) is the first (second) photon echo.

Figure S2 shows numerical calculations of the conventional two-pulse photon echo in a two-level optical ensemble. Figure S2(a) is a typical pulse sequence composed of data A and rephasing R, whose pulse area Φ_j $\left(\Phi_j = \int \Omega_j dt\right)$ is Φ_A=π/2 and Φ_R=π. Figures S2(b)~(h) are the results of Fig. S2(a), where Fig. S2(i) and (j) are for a double rephasing echo with an additional π–rephaisng pulse RR at t=12 μs. Figure S2(b) is the sum coherence evolution, representing a conventional photon echo. Due to the π–rephasing, the coherence of $Im\rho_{13}$ is inverted, resulting in emissive coherence. The photon echo also represents the result of time reversed coherence process by the π–rephasing pulse R (see Figs. S2(c) and (d)). The evolution speed of individual coherence ρ_{13} is predetermined by its detuning δ_j from the spectral line center between the ground state $|1\rangle$ and excited state $|3\rangle$. Thus, the overall coherence ρ_{13} is rapidly dephased out proportional to the inverse of the inhomogeneous width as shown in the first curve in Fig. S2(b). The novelty of rephasing is in the reversibility of the individual coherence evolutions, resulting in a macroscopic coherence burst, the so-called a photon echo (see the echo in Fig. S2(b)). Figure S2(c)~(f) show individual coherence evolutions for (a).

Figures S2(g) and (h) are for a particular detuned atom in Figs. S2(c) and (d), respectively. In Figs. S2(g) and (h), the imaginary term $Im\rho_{13}$ experiences a coherence inversion by R, whereas the real term $Re\rho_{12}$ has no sign change. Thus, the function of rephasing π–pulse can be expressed by $\rho_{13} \xrightarrow{R(\pi)} (\rho_{13})^*$. Although each real term oscillates sinusoidally with a π/2 phase shift with respect to the imaginary term and vice versa, the sum of symmetric real terms is always zero at any time.

The rephasing in Fig. S2 can also be expressed by the phase evolution of harmonic oscillation as following:

$$e^{+i\delta_j t} \xrightarrow{R(\pi)} e^{-i\delta_j T} e^{+i\delta_j t'} \left(= e^{+i\delta_j (t-2T)}\right), \qquad \text{(S-1)}$$

where $\rho(t) = \rho(0)e^{\pm i\delta_j t}$. The t' is the time after R, and T is the delay of R from A. Here, the sign reversal in the exponent is due to the rephasing. Thus, the photon echo is generated at t=2T to satisfy the maximum coherence as shown in Fig. S2(b).

In Figs. S2(i) and (j), a doubly rephased echo is shown as a result of an additional rephasing pulse RR at t=12 μs. Here, the first echo e1 is assumed not to affect the 2nd echo e2 [18,19,23,29]. The analytical expression of the double rephasing is given by:

$$\rho_{13} \xrightarrow{R(\pi)} (\rho_{13})^* \xrightarrow{RR(\pi)} \rho_{13}. \qquad \text{(S-2)}$$

As shown in Fig. S2(i), the doubly rephased echo e2 is obviously absorptive. Figure S2(j) is intentionally plotted for the oppositely detuned atom ($-\delta_j$), where the evolution direction is opposite to that in Fig. S2(h). The phase evolution of Fig. S2(j) is expressed by:

$$e^{-i\delta_j t} \xrightarrow{R(\pi)} e^{+i\delta_j T} e^{-i\delta_j t'} \xrightarrow{RR(\pi)} e^{-i\delta_j 2T} e^{-i\delta_j T'} e^{+i\delta_j t''} \left(= e^{+i\delta_j \left(t-2(T+T')\right)}\right), \qquad \text{(S-3)}$$

where $t' = t - T$ and $t'' = t - (2T + T')$. T and T' are 4 μs and 3 μs, respectively. Thus, the second echo e2 is generated at $t = t_A + 2(T + T') = 15\,\mu s$ as shown in Fig. S2(i), where t_A=1 μs. This doubly rephased photon echo obviously cannot be extracted from the medium due to its absorptive coherence.
